# Factory outbreak of Escherichia coli O157:H7 infection in Japan.

**DOI:** 10.3201/eid0503.990313

**Published:** 1999

**Authors:** Y Watanabe, K Ozasa, J H Mermin, P M Griffin, K Masuda, S Imashuku, T Sawada

**Affiliations:** Kyoto Prefectural University of Medicine, Kyoto, Japan. watanabe@basic.kpu-m.ac.jp

## Abstract

To determine the cause of a July 1996 outbreak of Escherichia coli O157:H7 among factory workers in Kyoto, Japan, we conducted cohort and case-control studies. Eating radish sprout salad during lunch at the factory cafeteria had been linked to illness. The sprouts were traced to four growers in Japan; one had been associated with an outbreak of E. coli O157:H7 among 6,000 schoolchildren in Sakai earlier in July.


### Factory Outbreak of *Escherichia coli* O157:H7 Infection in Japan

**Yoshiyuki Watanabe,* Kotaro Ozasa,* Jonathan H. Mermin,† Patricia M. Griffin,† Kazushige Masuda,‡ Shinsaku Imashuku,§ and Tadashi Sawada***
*Kyoto Prefectural University of Medicine, Kyoto, Japan;
†Centers for Disease Control and Prevention, Atlanta, Georgia, USA;
‡Public Health Bureau, Kyoto City Government, Kyoto, Japan; and
§Kyoto City Institute of Health and Environmental Sciences, Kyoto, Japan

To determine the cause of a July 1996 outbreak of *Escherichia coli* O157:H7 among factory workers in Kyoto, Japan, we conducted cohort and case-control studies. Eating radish sprout salad during lunch at the factory cafeteria had been linked to illness. The sprouts were traced to four growers in Japan; one had been associated with an outbreak of *E. coli* O157:H7 among 6,000 schoolchildren in Sakai earlier in July.

During May through August 1996, approximately 10,000 cases of *Escherichia coli* O157:H7 infection associated with at least 14 separate clusters were reported in Japan (1,2). Most cases occurred in school-age children. One cluster was a large outbreak in Sakai City, Osaka Prefecture, involving more than 6,000 primary school children. The outbreak started on July 13, 1996, and an investigation suggested that radish sprouts were the most likely cause (2,3).

An outbreak also occurred in a factory in Kyoto, approximately 50 km from Sakai City. On July 17, 1996, a 24-year-old male factory worker went to a local clinic with diarrhea. The next day, a second worker came to the clinic with diarrhea. Bloody diarrhea and hemolytic uremic syndrome (HUS) subsequently developed in both patients, and stool cultures from each yielded *E. coli* O157:H7. On July 21, a third worker died of HUS-associated encephalopathy; his stool culture later yielded *E. coli* O157:H7. All three workers had recently eaten meals at the factory cafeteria. To identify a possible food vehicle, we conducted an epidemiologic investigation.

Address for correspondence: Yoshiyuki Watanabe, Department of Social Medicine and Cultural Sciences, Research Institute for Neurological Diseases and Geriatrics, Kyoto Prefectural University of Medicine, Kawaramachi-Hirokoji, Kamigyo-ku, Kyoto 602-8566, Japan; fax: 81-75-251-5770; e-mail: watanabe@basic.kpu-m.ac.jp.

### The Study

On July 19, factory officials requested that ill workers report to factory health-care workers any symptoms from the beginning of July. Stool samples from workers with diarrhea were cultured for *E. coli* O157:H7 and other bacterial pathogens (e.g., *Salmonella* and *Shigella*). Surveillance continued until the end of July. A culture-confirmed case was defined as a stool culture yielding *E. coli* O157:H7 from a factory worker who had onset of diarrhea during July 15 to 22, 1996. A clinical case was defined as diarrhea with one or more loose stools per day with onset during July 15 to 22, 1996.

During their shifts, workers could eat any of the meals served at the factory cafeteria, which was operated by an outside company. Data on the date, time, and type of meal purchased at the cafeteria were routinely recorded by computer, and the cost of meals was deducted from employees' salaries. Workers could not pay by cash. We analyzed these data for visits to the cafeteria during July 8 to 14 (2 to 8 days before the date of symptom onset for the first case of culture-confirmed infection). All factory workers were included in analyses implicating a specific date of eating at the cafeteria. Only factory workers who purchased food at the cafeteria on a particular day were included in analyses of a particular meal for that day.

Two set lunches with prespecified food items were served for the same price in the cafeteria each day. Because the lunches could not be distinguished by computer records, a self-administered questionnaire was completed during September 24 to 27, 1996, by the 47 workers who had reported diarrhea and 300 randomly selected workers who had eaten at the factory cafeteria on the suspected exposure days (July 11 or 12) and had not reported diarrhea in July. A computer record of the meals purchased by each of the workers was included with each questionnaire to assist with recall.

Published methods were used to calculate odds ratios (ORs), 95% confidence intervals (CIs), and p values (4). P values were calculated by a chi-square test: p values of <0.05 were considered significant, and those of 0.05 to 0.09 were considered borderline significant. Multivariate conditional logistic regression analysis was conducted with Statistical Analysis System (SAS) software (SAS Institute, Cary, North Carolina, USA, 1990).

After reports of the first three cases, fecal samples from ill factory workers were cultured in sorbitol indole pyruvic acid bile salts agar (SIB) medium at 35° C to 37° C for 18 to 24 hours at the Kyoto City Institute of Health and Environmental Sciences. To differentiate *E. coli* O157:H7 from other bacteria, colonies were examined on triple sugar iron agar, sulfide indole motility medium, lysin indole motility semisolid agar, Voges-Proskauer semisolid medium, and Simon's citrate agar. Cultures that conformed to the biochemical pattern of *E. coli* O157:H7 were then serotyped. The presence of Shiga toxin 1 or 2 was confirmed by reversed passive latex agglutination and polymerase chain reaction (PCR). Stored food samples were homogenized, and a portion was cultured in modified *E. coli* broth before culturing in SIB medium. To differentiate strains of *E. coli* O157:H7, pulsed-field gel electrophoresis (PFGE) and random amplified polymorphic DNA-PCR (RAPD) assays were performed as previously described (5,6).

On July 18, the regional public health center examined the factory cafeteria kitchen facilities for deficiencies. All 25 food handlers were asked questions regarding abdominal symptoms and provided stool samples for bacteriologic testing. All food served was traced to the distributor and grower as far back as possible.

### Findings

Of the 3,155 employees of the factory, 74 reported gastrointestinal symptoms in July; stool samples were obtained from these workers. Illness in 47 persons met the case definition: 42 cases were clinically defined, and 5 were culture-confirmed. The peak date of symptom onset was July 17 (Figure). Six workers had only abdominal pain, fever, or general fatigue, and 21 had onset of diarrhea outside the defined period. HUS developed in three workers with culture-confirmed *E. coli* O157:H7 infection; two fully recovered; one died. One clinical case-patient and four culture-confirmed case-patients had bloody diarrhea. The proportion of cases with bloody diarrhea was 11% among all patients. The median age of case-patients was 30 years (18 to 61). Of the 47 case-patients, 45 (96%) (including all culture-confirmed cases) had eaten at the factory cafeteria during July 8 to 14. Of the 47 case-patients, 39 (83%) were male, and eight (17%) were female. No information on sex and age of the other factory workers was available.

Because the five workers with culture-confirmed *E. coli* O157:H7 infection had no common eating exposure except the factory cafeteria, we first analyzed the association between illness and date of eating at the cafeteria. Eating in the cafeteria any day during July 8 to 13 was associated with illness by univariate analysis. On multivariate logistic regression analysis, this association was significant or borderline significant only for July 11, 12, and 13. The ORs (95% CI, p value) of eating on July 11, 12, and 13 were 2.58 (0.91 to 7.36, 0.08),

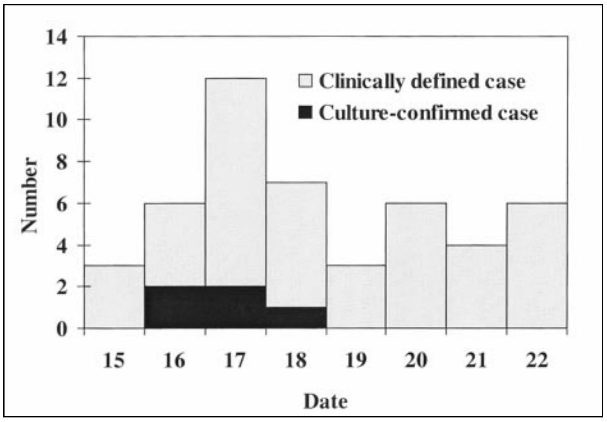

Figure. *Escherichia coli* O157:H7 infection by date of symptom onset, July 15-21, 1996.

2.84 (1.02 to 7.94, 0.05), and 3.19 (1.03 to 9.86, 0.04), respectively. Because 81% of the patients ate in the cafeteria on July 11 and 12 compared with 23% on July 13, July 11 and 12 were considered the most likely days of exposure. On multivariate analysis of the six meal times on July 11 and 12, only eating lunch in the cafeteria on July 11 was associated with illness. The rate of diarrhea for 1,134 workers who ate lunch on July 11 was 3.0%, compared with 0.6% for 2,021 workers who did not (OR = 3.04, 95% CI = 1.08, p = 0.04).

Of the 47 case-patients, 44 (94%) responded to the questionnaire. In 31 patients who answered the question regarding symptoms, diarrhea lasted for a median of 3 days (1 to 10 days), and four (13%) reported bloody diarrhea. Of 300 potential controls randomly selected from factory workers who ate in the cafeteria on July 11 or 12, 291 (97%) responded to the questionnaire. Among the respondents, 16 (5%) reported gastrointestinal symptoms in July (of these, four [25%] had diarrhea during July 15-22, but none reported bloody diarrhea), and three did not respond to the question regarding symptoms; 272 respondents who reported no illness were adopted as controls. The median age was similar for case-patients (30 years [18 to 61 years]) and controls (32 years [20 to 65 years]). Eighty percent of case-patients and 83% of controls were male.

Among the participants, 29 patients and 164 controls responded that they clearly remembered if they had eaten the radish sprout salad. Seventeen (59%) of 29 patients and 64 (39%) of 164 controls reported eating radish sprout salad (OR = 2.21, 95% CI = 0.99 to 4.94, p = 0.08) (Table). No other food item served on July 11 or 12 was eaten by >50% of patients and had a higher odds ratio than radish sprout salad. Among the five patients with culture-confirmed infection, computer records indicated that four patients, including the one who died, ate radish sprouts. Radish sprout salad (consisting of radish sprouts, mayonnaise, cauliflower, and fish paste) was served with both lunches the cafeteria served on July 11, the only time sprouts were served during July 8 to 14.

All five patient isolates of *E. coli* O157:H7 produced Shiga toxins 1 and 2. *E. coli* O157:H7 was not detected in any of the frozen food samples (including radish sprout salad) leftover from cafeteria meals during July 11 to 15. Both PFGE and RAPD patterns of the *E. coli* O157:H7 isolates from this outbreak and the outbreak in Sakai City during the same time were indistinguishable (1-3,7).

Examination of the factory cafeteria kitchen facilities on July 18 by the regional public health center found no deficiencies. One female food handler had diarrhea with onset July 17, but *E. coli* O157:H7 was not cultured from her stool specimen or from specimens of any of the other food handlers.

The radish sprouts served at the cafeteria on July 11 were supplied by a single distributor that received the sprouts from four growers, one of whom also supplied the radish sprouts suspected as the source of *E. coli* O157:H7 infections in the Sakai City school outbreak. Radish sprouts used at the primary schools in Sakai City and at the factory cafeteria had been shipped by the grower on July 9 (3); however, the sprouts used at the factory cafeteria had been purchased along with radish sprouts from different growers.

### Conclusions

Our data indicate that the outbreak of *E. coli* O157:H7 infection among Kyoto factory workers was most likely caused by contaminated radish sprouts: the factory outbreak began during the week following the Sakai City outbreak; the factory used radish sprouts from the same grower; they were shipped on the same day as those served to school children in the Sakai City outbreak; and isolates from both outbreaks had indistinguishable PFGE and RAPD patterns (1-3,5,7). The PFGE patterns of earlier outbreaks in Okayama Prefecture (Oku-cho), Gifu Prefecture, Hiroshima Prefecture, Aichi

Table. Factory cafeteria foods associated with illness, July 11, 1996, Kyoto, Japan

| Food | Case-patients exposed/total(%) | Controls exposed/total (%) | Odds ratio[a] (95% CI) | p value |
|---|---|---|---|---|
| Radish sprout salad | 17/29(58.6) | 64/164(39.0) | 2.21(0.99-4.94) | 0.08 |
| Boiled beef with soy sauce | 8/28(28.6) | 24/152(15.8) | 2.13(0.84-5.40) | 0.18 |
| Scrambled eggs | 10/28(35.7) | 31/150(20.7) | 2.11(0.89-5.04) | 0.18 |

[a]Odds ratio>2.00; CI: confidence interval.

Prefecture, and Okayama Prefecture (Niimi City) were indistinguishable from each other and different from the PFGE patterns of isolates from the outbreaks in Sakai City and the Kyoto factory (1-3,5). *E. coli* O157:H7 was not isolated from radish sprouts; however, the process of freezing sprouts or pooling them with other food items may have decreased the number of organisms to an undetectable level.

Although radish sprouts had never been linked to *E. coli* O157:H7 infection, they are plausible vehicles. Most outbreaks of *E. coli* O157:H7 infections have been linked to ground beef (8), but other items, including unrefrigerated sandwiches (9), apple cider (10), mayonnaise (11), cantaloupe (12), lettuce (13), and alfalfa sprouts (14,15) have been implicated. In addition, some sprout types, including alfalfa sprouts (16) and mung bean sprouts (17), have been linked to *Salmonella* outbreaks. In 1997, *E. coli* O157:H7 was isolated from radish sprouts collected from two different outbreaks of *E. coli* O157:H7 infections in Japan (1).

Three cases of HUS (6%) among 47 cases of clinically or laboratory-defined cases of *E. coli* O157:H7 infection in the factory outbreak is comparable to rates described in other outbreaks (18,19). The proportion of workers reporting bloody diarrhea was low, possibly because infection with *E. coli* O157:H7 follows a more benign course in adults than in children (20) or because the amount of bacterial contamination was low.

Several reasons might explain the small proportion of workers who ate lunch on July 11 and reported illness. First, some ill workers might not have informed the factory health-care personnel about gastrointestinal symptoms for fear of decreasing their chance for future promotion. This seems plausible because 16 (5%) of 291 potential controls in the case-control study mentioned unreported gastrointestinal symptoms. If this percentage of underreporting occurred for the 3,155 workers in the factory, an additional 173 infections may have been missed. Second, the pathogens might have been diluted because only a part of the radish sprout shipment was contaminated. The latter hypothesis is supported by the fact that four growers, including the one implicated in the Sakai City outbreak, supplied the radish sprouts eaten at the factory cafeteria on July 11. Third, the contamination of radish sprouts may have been reduced by washing. Although the association between eating radish sprout salad and illness among workers who ate lunch on July 11 was of borderline significance, it was the only item associated with illness that was consumed by more than 50% of case-patients. Moreover, the next lowest p value was 0.18, far from that of radish sprout salad.

The radish sprout salad contained other food items; therefore, the individual risk for each food item could not be ascertained. However, radish sprouts and mayonnaise were the only uncooked ingredients. Although mayonnaise is a possible vehicle, no reports implicated it in other outbreaks in Japan in 1996 (1).

Recall bias could have occurred in the case-control study because workers were asked about meals they had eaten 8 weeks earlier. Providing with the questionnaire a printout of food items purchased on July 11 and 12 may have assisted recall. In addition, as a result of the outbreak, the cafeteria stopped serving food on July 19 and had not resumed service at the time of the case-control study. This may have assisted respondents in remembering what food items they had eaten during the last week of dining in the cafeteria.

### Acknowledgments

The authors thank the workers and staff of the Kyoto factory where the outbreak occurred for their cooperation in the survey; Drs. Yurie Kanamoto, Hiroyuki Imai, Shouji Tateishi, Mitsuaki Nishibuchi, Jiro Imanishi, Jun Fukuda, Yusaku Matsui, Yoichi Mori, and Kozo Yokota, and collaborators in the Kyoto City Institute of Health and Environmental Sciences; Mr. Akio Kuroda and Ms. Hisae Takenobu; and Drs. Robert Hyams and Robert Tauxe for helpful discussions and for facilitating the investigation.

Dr. Watanabe is professor, Department of Social Medicine and Cultural Sciences, Research Institute for Neurological Diseases and Geriatrics, Kyoto Prefectural University of Medicine. His research focuses on the epidemiology of gastrointestinal and neurologic diseases and geriatrics.

### References

1. National Institute of Health and Infectious Diseases Control Division, Ministry of Health and Welfare of Japan. Verocytotoxin-producing *Escherichia coli* (enterohemorrhagic *E. coli*) infections, Japan, 1996-June 1997. Infectious Agents Surveillance Report 1997;18:153-4.
2. National Institute of Health and Infectious Diseases Control Division, Ministry of Health and Welfare of Japan. Enterohemorrhagic *Escherichia coli* (verocytotoxin-producing *E. coli*) infection, 1996-April 1998. Infectious Agents Surveillance Report 1998;19:122-3.

3. Study report on the cause of the outbreak of diarrhea due to *E. coli* O157:H7 among primary school students in Sakai City. Tokyo: Ministry of Health and Welfare in Japan, Environmental Health Bureau, Food Sanitation Division; 1996. (In Japanese).
4. Rothman KJ. Modern epidemiology. Boston: Little, Brown and Company; 1986. p. 153-76.
5. Watanabe H, Wada A, Inagaki Y, Itoh K, Tamura K. Outbreaks of enterohaemorrhagic *Escherichia coli* O157:H7 infection by two different genotype strains in Japan, 1996. Lancet 1996;348:831-2.
6. Izumiya H, Terajima J, Wada A, Inagaki Y, Itoh K, Tamura K, et al. Molecular typing of *Escherichia coli* O157:H7 isolates in Japan by using pulsed-field gel electrophoresis. J Clin Microbiol 1997;35:1675-80.
7. Report on the cause of the outbreak of *E. coli* O157:H7 in Kyoto City in 1996. Kyoto, Japan: Public Health Bureau, Kyoto City Government; 1997. (In Japanese).
8. Boyce TG, Swerdlow DL, Griffin PM. *E. coli* O157:H7 and the hemolytic-uremic syndrome. N Engl J Med 1995;333:364-8.
9. Carter AO, Borczyk AA, Carlson JA, Harvey B, Hockin JC, Karmali MA, et al. A severe outbreak of *Escherichia coli* O157:H7-associated hemorrhagic colitis in a nursing home. N Engl J Med 1987;317:1496-500.
10. Besser RE, Lett SM, Weber JT, Doyle MP, Barrett TJ, Wells JG, et al. An outbreak of diarrhea and hemolytic uremic syndrome from *Escherichia coli* O157:H7 in fresh-pressed apple cider. JAMA 1993;269:2217-20.
11. Keene WE, Mcanulty JM, Williams LP, Hoesly FC, Hedberg K, Fleming DW, et al. A two-restaurant outbreak of *Escherichia coli* O157:H7 enteritis associated with consumption of mayonnaise [abstract]. In: Proceedings of the 33rd Interscience Conference on Antimicrobial Agents and Chemotherapy; 1993 Oct 17-20; New Orleans, Louisiana. Washington: American Society for Microbiology; 1993. p. 354.
12. Yet another outbreak of hemorrhagic colitis—Corvallis. Portland (OR): Center for Disease Prevention and Epidemiology, Oregon Health Division, Department of Human Resources. CD Summary 1993;42:1-2.
13. Mermin JH, Hilborn ED, Voetsch A, Swartz M, Lambert-Fair MA, Farrar J, et al. A multistate outbreak of *Escherichia coli* O157:H7 infections associated with eating mesclum mix lettuce [abstract]. In: Proceedings of the 3rd International Symposium and Workshop on Shiga Toxin (verocytotoxin)-producing *Escherichia coli* infections; 1997 Jun 22-26; Baltimore, Maryland. The Lois Joy Galler Foundation; 1997. p. 9.
14. Outbreaks of *Escherichia coli* O157:H7 infection associated with eating alfalfa aprouts—Michigan and Virginia, Jun-Jul 1997. MMWR Morb Mortal Wkly Rep 1997;46:741-4.
15. Mohle-Boetani JC, Werner SB, Farrar JA, Abbott S, Bryant R, Vugia DJ. Outbreak of toxigenic *E. coli* O157:H7: non-motile (NM) associated with a clover-alfalfa mix [abstract]. In: Program and Abstracts of the Infectious Diseases Society of America 36th Annual Meeting; 1998 Nov 12-15; Denver, Colorado. 536 Fr, 178.
16. Mahon BE, Ponka A, Hall WN, Komatsu K, Dietrich SE, Siitonen A, et al. An international outbreak of *Salmonella* infections caused by alfalfa sprouts grown from contaminated seeds. J Infect Dis 1997;175:876-82.
17. O'Mahoney M, Cowden J, Smyth B, Lynch D, Hall M, Rowe B, et al. An outbreak of *Salmonella saint-paul* infections associated with bean sprouts. Epidemiol Infect 1990;104:229-35.
18. Bell BP, Goldoft M, Griffin PM, Davis MA, Gordon DC, Tarr PI, et al. A multistate outbreak of *Escherichia coli* O157:H7–associated bloody diarrhea and hemolytic uremic syndrome from hamburgers: the Washington experience. JAMA 1994;272:1349-53.
19. Akashi S, Joh K, Tsuji A, Ito K, Hoshi H, Hayakawa T, et al. A severe outbreak of haemorrhagic colitis and haemolytic uraemic syndrome associated with *Escherichia coli* O157:H7 in Japan. Eur J Pediatr 1994;153:650-5.
20. Rodrigue DC, Mast EE, Greene KD, Davis JP, Hutchinson MA, Wells JG, et al. A university outbreak of *Escherichia coli* O157:H7 infection associated with roast beef and an unusually benign clinical course. J Infect Dis 1995;172:1122-5.

